# Lung ultrasound score and in-hospital mortality of adults with acute respiratory distress syndrome: a meta-analysis

**DOI:** 10.1186/s12890-023-02826-5

**Published:** 2024-01-29

**Authors:** Dandan Wang, Yun Qi

**Affiliations:** 1 Department of Ultrasound, Haikou Affiliated Hospital of Central South University Xiangya School of Medicine, 570311 Haikou, China; 2 Department of Emergency Medicine, Haikou Affiliated Hospital of Central South University Xiangya School of Medicine, No. 43 Renmin Dadao, Meilan District, 570311 Haikou, China

**Keywords:** Acute respiratory distress syndrome, Lung ultrasound score, Prognosis, Mortality, Meta-analysis

## Abstract

**Background:**

Lung ultrasound (LUS) score could quantitatively reflect the lung aeration, which has been well applied in critically ill patients. The aim of the systematic review and meta-analysis was to evaluate the association between LUS score at admission and the risk of in-hospital mortality of adults with acute respiratory distress syndrome (ARDS).

**Methods:**

Toachieve the objective of this meta-analysis, we conducted a thorough search of PubMed, Embase, Cochrane Library, and the Web of Science to identify relevant observational studies with longitudinal follow-up. We employed random-effects models to combine the outcomes, considering the potential influence of heterogeneity.

**Results:**

Thirteen cohort studies with 1,022 hospitalized patients with ARDS were included. Among them, 343 patients (33.6%) died during hospitalization. The pooled results suggested that the LUS score at admission was higher in non-survivors as compared to survivors (standardized mean difference = 0.73, 95% confidence interval [CI]: 0.55 to 0.91, p < 0.001; I^2^ = 25%). Moreover, a high LUS score at admission was associated with a higher risk of in-hospital mortality of patients with ARDS (risk ratio: 1.44, 95% CI: 1.14 to 1.81, p = 0.002; I^2^ = 46%). Subgroup analyses showed consistent results in studies with LUS score analyzed with 12 or 16 lung regions, and in studies reporting mortality during ICU or within 1-month hospitalization.

**Conclusion:**

Our findings suggest that a high LUS score at admission may be associated with a high risk of in-hospital mortality of patients with ARDS.

## Introduction

Acute respiratory distress syndrome (ARDS) is a severe pathological state characterized by refractory hypoxemia resulting from diverse etiological factors, commonly arising as a secondary manifestation of pulmonary disorders or extrapulmonary conditions, including pneumonia, drowning, and non-pulmonary sepsis [1–3]. Clinically, ARDS has been associated with a multitude of detrimental outcomes, encompassing respiratory failure, critical illness, and fatality [4]. Among patients admitted to the intensive care unit (ICU), the incidence of ARDS has been estimated to approximate 10% [5]. Despite advancements in intensive care over the past few decades, ARDS remains an underrecognized, deleterious, and potentially fatal ailment, with mortality rates reaching as high as 50% [6]. Consequently, it is imperative to ascertain the risk factors associated with immediate mortality in individuals afflicted with ARDS.

Lung ultrasound (LUS) has emerged as a valuable and non-invasive modality for promptly assessing chest conditions in individuals with respiratory disorders, particularly in acute scenarios like emergency department and ICU settings [7, 8]. The growing body of evidence indicates that LUS offers several advantages, including cost-effectiveness, expeditiousness, absence of ionizing radiation, bedside accessibility, and method repeatability [9, 10]. In patients with ARDS, the evaluation of lung aeration can be accomplished by monitoring alterations in the appearance of A-lines and B-lines across various lung regions, and quantitatively evaluated as the LUS score [11]. A recent comprehensive study has substantiated the precision of the LUS aeration score in diagnosing ARDS [12, 13]. Nevertheless, preliminary studies have yielded incongruous findings concerning the correlation between the LUS score upon admission and the likelihood of in-hospital mortality among adult patients with ARDS [14–26]. Given this prevailing uncertainty, we conducted a systematic review and meta-analysis to consolidate the present comprehension of the prognostic significance of the LUS score upon admission for ARDS.

## Materials and methods

Throughout the process of planning, conducting, and reporting the study, the Preferred Reporting Items for Systematic Reviews and Meta-Analyses statement [27, 28] and Cochrane Handbook [29] were followed.

### Inclusion and exclusion criteria of studies

Inclusion criteria were developed per PICOS recommendations and according to the aim of the meta-analysis.

P (patients): Hospitalized adult patients (18 years or older) with a confirmed diagnosis of ARDS, which was diagnosed in accordance with the Berlin Definition [1].

I (exposure): LUS score was measured at admission. Methods for measuring LUS score were consistent with the protocols used among the included studies, which generally included the protocols of 12-region method (0 ~ 36 points of score) and a 16-region method (0 ~ 48 points) [30, 31]. Patients with a high LUS score at admission were considered as exposure, and the cutoff for defining high LUS score was also according to the cutoff used among the original studies.

C (control): Patients with a low LUS score at admission.

O (outcomes): Incidence in all-cause mortality during hospitalization was observed. The primary outcome was to compare the difference of LUS score at baseline between non-survivors and survivors of hospitalized patients with ARDS. The secondary outcome was to compare the incidence of in-hospital mortality between hospitalized ARDS patients with high versus low LUS score at admission.

S (study design): Studies with longitudinal follow-up, including cohort and nested case-control studies, and post-hoc analyses of clinical trials. Excluded from the meta-analysis were reviews, editorials, preclinical studies, and studies that did not involve patients with ARDS, studies including neonates or children, studies did not measure LUS score at baseline, or studies did not report the outcome of in-hospital mortality. In instances with a patient population overlap, the study with the greatest sample size was incorporated into the meta-analysis.

### Search of databases

We searched electronic databases, including PubMed, Embase, Cochrane Library, and Web of Science, starting from inception and ending July 21, 2023, for studies that had been published up to that date. The search was performed with terms related to our study, including (1) “ultrasonography” OR “ultrasound” OR “ultrasonic” OR “ultrasonographic”; (2) “lung” OR “pulmonary”; (3) “score” OR “scores”; and (4) “acute respiratory distress syndrome” OR “respiratory distress syndrome” OR “ARDS”. Only studies published as full-length articles in English or Chinese peer-reviewed journals were considered. As part of our manual screening process, references from relevant original and review articles were screened for possible relevant studies.

### Data extraction and quality evaluation

Two authors independently conducted comprehensive literature searches, collected data, and assessed the quality of the included studies. In cases where discrepancies arose, discussions between the two authors were indicated until a consensus was achieved. The analysis of the studies involved the extraction of relevant information, such as study details, design characteristics, patient diagnoses, demographic factors, methods and timing for LUS examination, follow-up duration, number of patients that died during hospitalization, outcomes reported, and the variables matched or adjusted for evaluating the association between LUS score and in-hospital mortality of patients with ARDS. To evaluate the quality of the included studies, we utilized the Newcastle–Ottawa Scale (NOS) [32], which assesses participant selection, comparability of the study groups, and the validity of the outcomes. The NOS scoring system comprises nine stars, with more stars indicating a higher quality study.

### Statistics

Standardized mean differences (SMD) and corresponding 95% confidence interval (CI) were used to present the potential difference of LUS score at admission between the non-survivors and survivors of patients with ARDS. Risk ratios (RR) and corresponding 95% CI were used as the variables to indicate the association between LUS score and the risk of in-hospital mortality among patients with ARDS. A logarithmical transformation was performed on the RR and its corresponding standard error from each study to stabilize and normalize its variance [33]. In order to estimate between-study heterogeneity, the Cochrane Q test and the I^2^ statistic [34] were used. An I^2^ > 50% indicates that there is significant heterogeneity between studies. A random-effects model was employed to amalgamate the findings, as it has been acknowledged to encompass the impact of potential heterogeneity [29]. Sensitivity analysis by excluding one study at a time was performed to evaluate the influence of individual study on the results of the meta-analyses. Subgroup analyses were also performed to evaluate the potential influence of study characteristics on the results, such as study design, methods and cutoffs for evaluating LUS score, and follow-up durations. A funnel plot and Egger’s regression asymmetry test were used to estimate publication bias based on visual symmetry judgments [35]. The statistical analyses were done with RevMan (Version 5.1; Cochrane Collaboration, Oxford, UK) and Stata software (version 12.0; Stata Corporation, College Station, TX).

## Results

### Database search and study retrieval

**Fig. 1:**
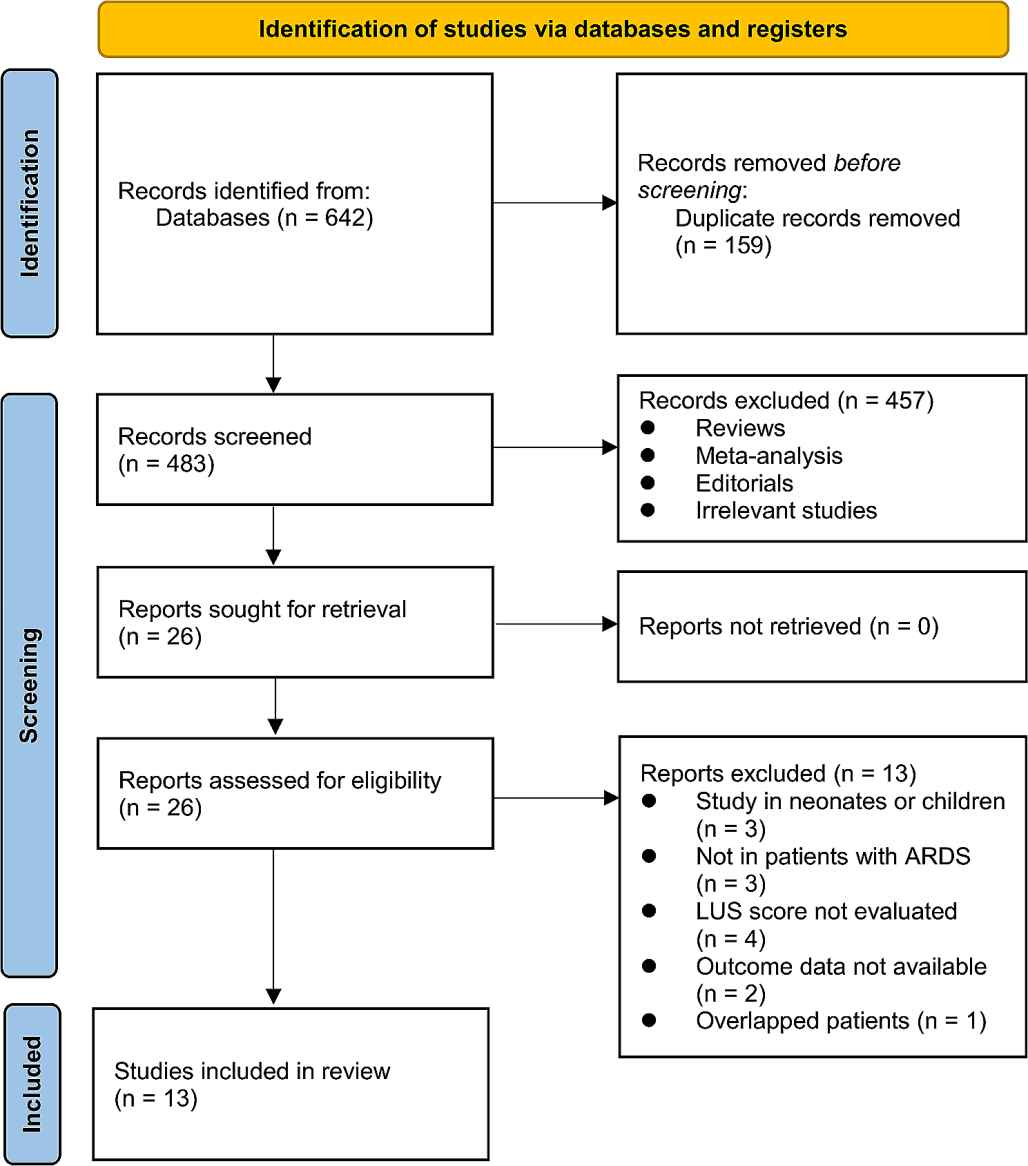
Flowchart of database search and study inclusion

Figure 1 illustrates the step-by-step process of the literature search and study retrieval. Initially, a total of 642 records were identified from the databases. After removing 159 duplicate entries, 483 unique records remained. Subsequently, 457 studies were excluded during the initial screening of titles and abstracts as they did not align with the objectives of the meta-analysis. After this initial screening, 26 studies were selected for further assessment through full-text reviews. Following the rigorous review process, 13 studies were excluded for specific reasons detailed in Fig. 1. Consequently, 13 cohort studies were deemed suitable and were included in the subsequent meta-analysis [14–26].

### Study characteristics

Overall, 13 cohort studies, including eight prospective studies [15, 17, 19, 20, 22–24, 26] and five retrospective studies [14, 16, 18, 21, 25], were analyzed in this meta-analysis. The characteristics of the included studies are summarized in Table 1. These studies were performed in China, India, and Italy, and published between 2014 and 2023. A total of 1,022 hospitalized adult patients with ARDS were included. The etiologies of ARDS varied among the included studies, with four studies included patients with ARDS related to infection [14, 17, 21, 23]. The mean ages of the patients were 51 to 78 years, and the proportions of men were 43 to 83%. For all the included studies, LUS was performed within 24 h after admission, with the 12-region method used in ten studies [15, 16, 19–26], and the 16-region method in three studies [14, 17, 18]. The follow-up duration was during ICU in six studies [15, 16, 21–23, 25], and was 28 or 30 days in seven studies [14, 17–20, 24, 26]. Among the included patients, 343 patients (33.6%) died during hospitalization. The difference of LUS score at baseline between non-survivors and survivors were reported in 11 studies [14–17, 19–21, 23–26], and RR of in-hospital mortality between patients with high versus low LUS score were reported in six studies [18, 20, 22, 23, 25, 26]. Median of LUS score was selected as the cutoff value in four studies [18, 20, 22, 23], while the receiver operating characteristic (ROC) analysis derived cutoff value was used in the other two studies [25, 26]. Univariate analysis was used to determine the association between LUS score and in-hospital mortality in two studies [14, 18], age was controlled in the remaining 11 studies [15–17, 19–26], with nine of them also controlled for other confounding factors [15, 19–26]. The NOS of the included studies were 6 to 9, indicating that they were of moderate to good quality (Table 2).

**Table 1:** Characteristics of the included studies

Study	Country	Design	Diagnosis	No. of patients included	Etiology	Infective etiology (%)	Mean age (years)	Men (%)	Timing of LUS	Methods for LUS analysis	Follow-up duration	No. of patients died	Outcomes reported	Variables matched or adjusted
Ding 2014	China	RC	Adults with ARDS on MV	29	Severe pneumonia, sepsis-related, or CTD with pulmonary infection	100	64.4	58.6	At ICU admission	16 regions, score: 0 ~ 48	28 days	9	Difference of LUS score	None
Zhao 2015	China	RC	Adults with ARDS on MV	21	Severe pneumonia, aspiration, sepsis, after CPR, or traumatic	76.2	78	67	Within 24 h after ICU admission	12 regions, score: 0 ~ 36	During ICU	13	Difference of LUS score	Age
Li 2015	China	PC	Adults with ARDS	62	NR	NR	67	43.6	At ICU admission	12 regions, score: 0 ~ 36	During ICU	27	Difference of LUS score	Age, sex, etiology, and disease severity
Wang 2016	China	PC	Adults with ARDS on MV	45	Severe pneumonia or septic shock	100	65	71.1	At ICU admission	16 regions, score: 0 ~ 48	28 days	12	Difference of LUS score	Age
Xie 2019	China	PC	Adults with ARDS on MV	83	NR	NR	60.3	63.9	At ICU admission	12 regions, score: 0 ~ 36	28 days	32	Difference of LUS score	Age, sex, BMI, and comorbidities
Lv 2019	China	RC	Adults with ARDS	112	NR	NR	60.3	63.9	At ICU admission	16 regions, score: 0 ~ 48	30 days	39	RR for death between high versus low LUS score (cutoff: median)	None
Zhang 2020	China	RC	Adults with ARDS	46	Severe pneumonia or septic shock	100	73.3	56.5	At ICU admission	12 regions, score: 0 ~ 36	During ICU	14	Difference of LUS score	Age, sex, LVEF, and HR
Yu 2020	China	PC	Adults with ARDS	116	Severe pneumonia, sepsis-related, or trauma-related	77.6	55.4	63.8	At admission	12 regions, score: 0 ~ 36	28 days	39	Difference of LUS score, and RR for death between high versus low LUS score (cutoff: median)	Age, sex, BMI, etiology, and comorbidities
Xie 2021	China	PC	Adults with ARDS on MV	121	NR	NR	62.8	60.3	At admission	12 regions, score: 0 ~ 36	28 days	46	Difference of LUS score	Age, sex, BMI, and comorbidities
Chaudhuri 2021	India	PC	Adults with ARDS	100	Severe pneumonia, sepsis-related, or other critical illness related	NR	51.2	63	At admission	12 regions, score: 0 ~ 36	During ICU	21	RR for death between high versus low LUS score (cutoff: median)	Age, sex, APACHE II score, SOFA score, PaO2/FiO2, and albumin
Lazzeri 2021	Italy	PC	Adults with COVID-related ARDS	47	COVID-19	100	63	82.9	At ICU admission	12 regions, score: 0 ~ 36	During ICU	13	Difference of LUS score, and RR for death between high versus low LUS score (cutoff: median)	Age, sex, and SPAP
Guo 2022	China	RC	Adults with ARDS	98	NR	NR	54.8	54.1	At ICU admission	12 regions, score: 0 ~ 36	During ICU	22	Difference of LUS score, and RR for death between high versus low LUS score (cutoff: ROC-carve analysis derived)	Age, sex, APACHE II score, and PaO2/FiO2
Zheng 2023	China	PC	Adults with ARDS	142	NR	NR	62.7	57	At ICU admission	12 regions, score: 0 ~ 36	28 days	56	Difference of LUS score, and RR for death between high versus low LUS score (cutoff: ROC-curve analysis derived)	Age, sex, and etiology

LUS, lung ultrasound; retrospective cohort; PC, prospective cohort; ARDS, acute respiratory distress syndrome; MV, mechanical ventilation; CTD, connective tissue disease; CPR, cardiopulmonary resuscitation; COVID-19, coronavirus disease 2019; NR, not reported; ICU, intensive care unit; RR, risk ratio; ROC, receiver operating characteristic; BMI, body mass index; LVEF, left ventricular ejection fraction; HR, heart rate; APACHE II, Acute Physiology and Chronic Health Evaluation II; SOFA, Sequential Organ-Failure Assessment; SPAP, simplified acute physiologic score;

**Table 2:** Study quality evaluation via the Newcastle-Ottawa Scale

Study	Representativeness of the exposed cohort	Selection of the non-exposed cohort	Ascertainment of exposure	Outcome not present at baseline	Control for age	Control for other confounding factors	Assessment of outcome	Enough long follow-up duration	Adequacy of follow-up of cohorts	Total
Ding 2014	0	1	1	1	0	0	1	1	1	6
Zhao 2015	0	1	1	1	1	0	1	1	1	7
Li 2015	1	1	1	1	1	1	1	1	1	9
Wang 2016	1	1	1	1	1	0	1	1	1	8
Xie 2019	1	1	1	1	1	0	1	1	1	9
Lv 2019	0	1	1	1	1	0	0	1	1	6
Zhang 2020	0	1	1	1	1	1	1	1	1	8
Yu 2020	1	1	1	1	1	1	1	1	1	9
Xie 2021	1	1	1	1	1	1	1	1	1	9
Chaudhuri 2021	1	1	1	1	1	1	1	1	1	9
Lazzeri 2021	1	1	1	1	1	1	1	1	1	9
Guo 2022	0	1	1	1	1	1	1	1	1	8
Zheng 2023	1	1	1	1	1	1	1	1	1	9

### Difference of LUS score between non-survivors and survivors

**Fig. 2:**
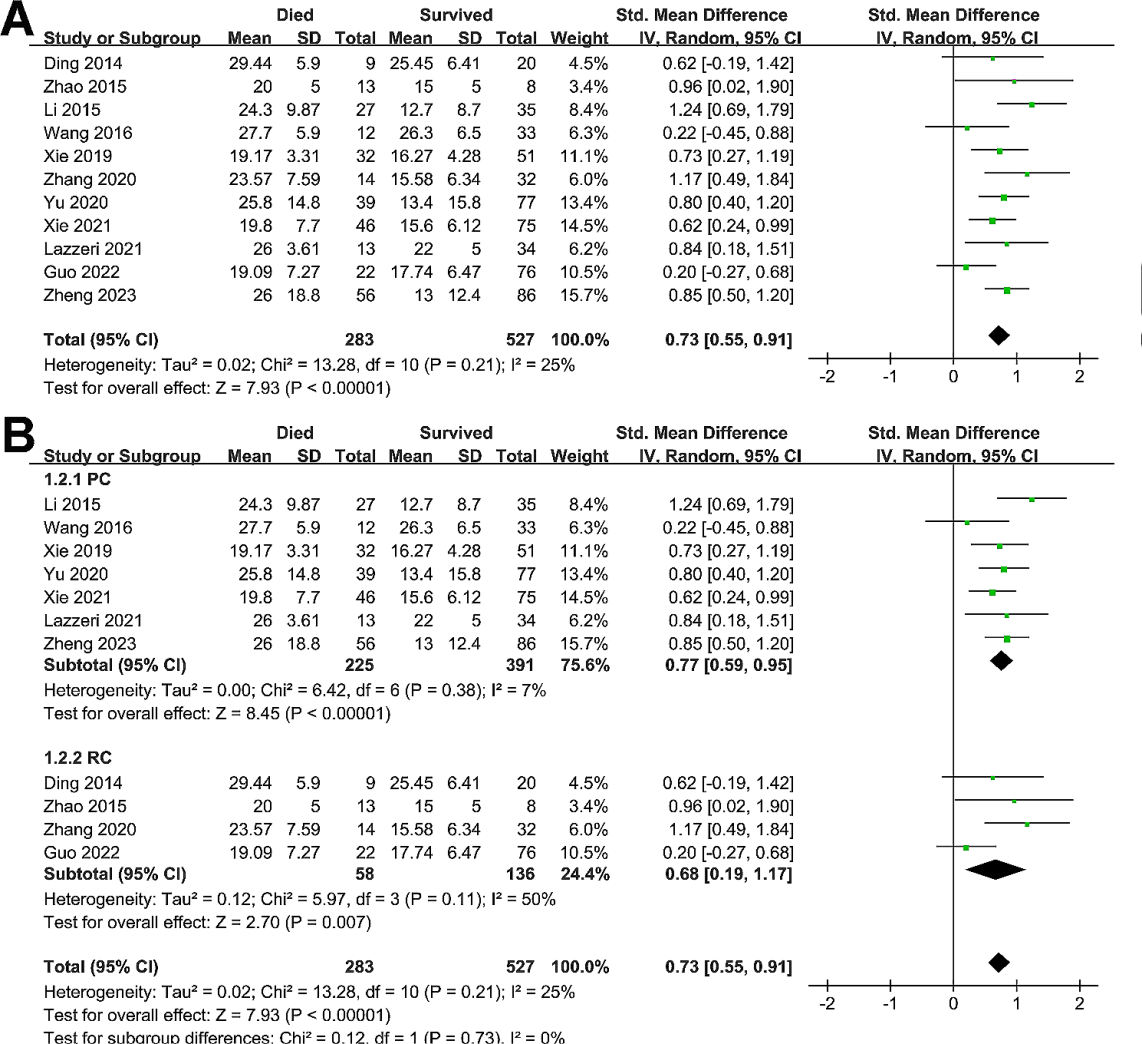
Forest plots for the meta-analysis of the difference of LUS score at admission between non-survivors and survivors of ARDS patients; **(A)**, overall meta-analysis; and **(B)**, subgroup analysis according to study design

**Fig. 3:**
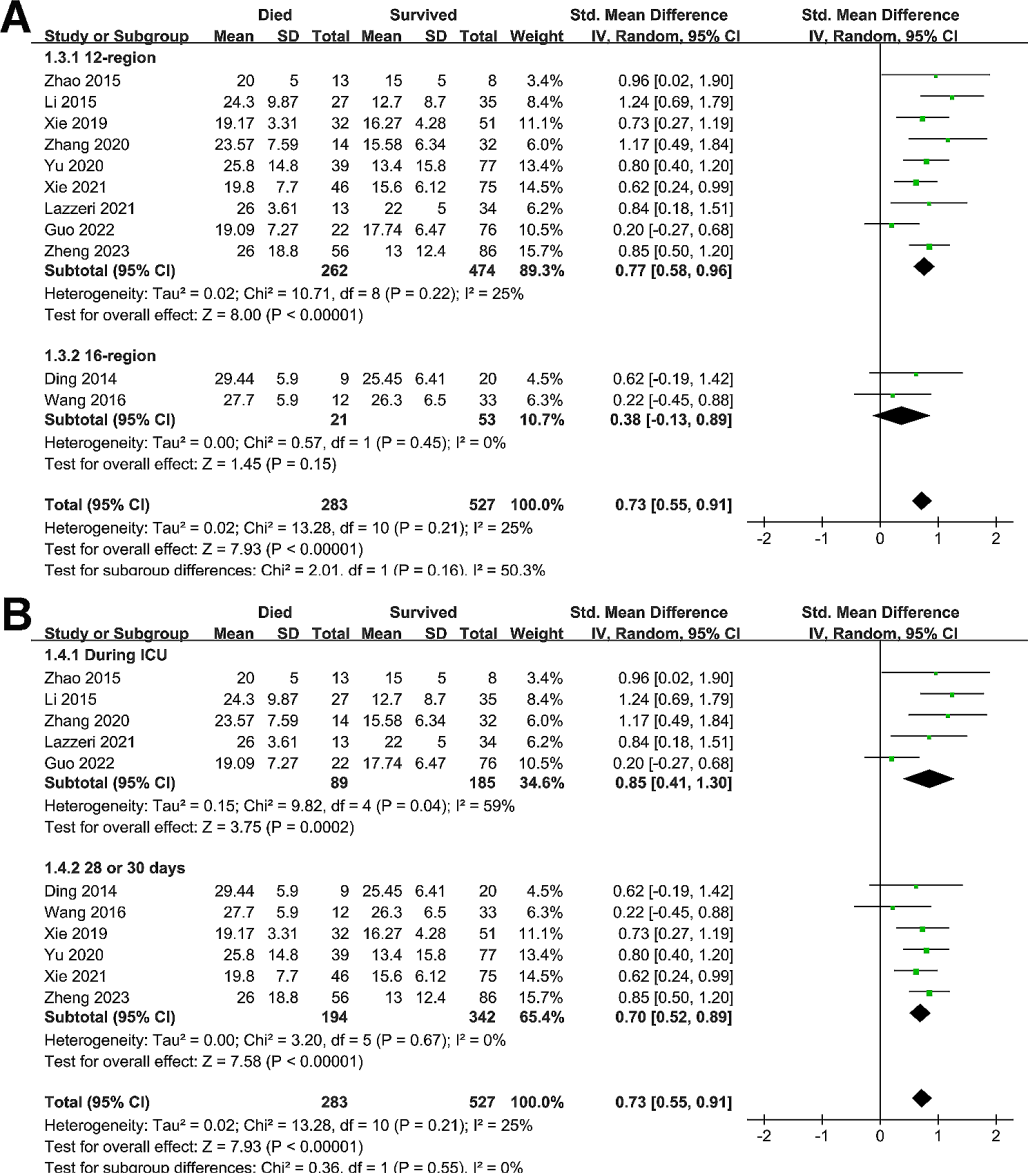
Forest plots for the meta-analysis of the difference of LUS score at admission between non-survivors and survivors of ARDS patients; **(A)**, subgroup analysis according to methods for evaluating LUS score; and **(B)**, subgroup analysis according to follow-up durations

Pooled results with 11 studies [14–17, 19–21, 23–26] showed that compared to survivors during hospitalization, patients with ARDS who died during hospitalization had a significant higher LUS score at admission (SMD = 0.73, 95% CI: 0.55 to 0.91, p < 0.001; Fig. 2A) with mild heterogeneity (I^2^ = 25%). Sensitivity analysis by excluding one study at a time showed consistent results (SMD: 0.69 to 0.76, p all < 0.05). Specifically, sensitivity analysis limited to studies of patients with infectious etiologies of ARDS showed similar results (SMD = 0.71, 95% CI: 0.31 to 1.12, p < 0.001; I^2^ = 27%). Further subgroup analyses showed consistent results in prospective and retrospective studies (p for subgroup difference = 0.73, Fig. 2B), in studies of LUS score analyzed with 12-region or 16-region method (p for subgroup difference = 0.16, Fig. 3A), and in studies reporting mortality during ICU or within 1-month hospitalization (p for subgroup difference = 0.55, Fig. 3B).

### RR for the association between LUS score and in-hospital mortality

**Fig. 4:**
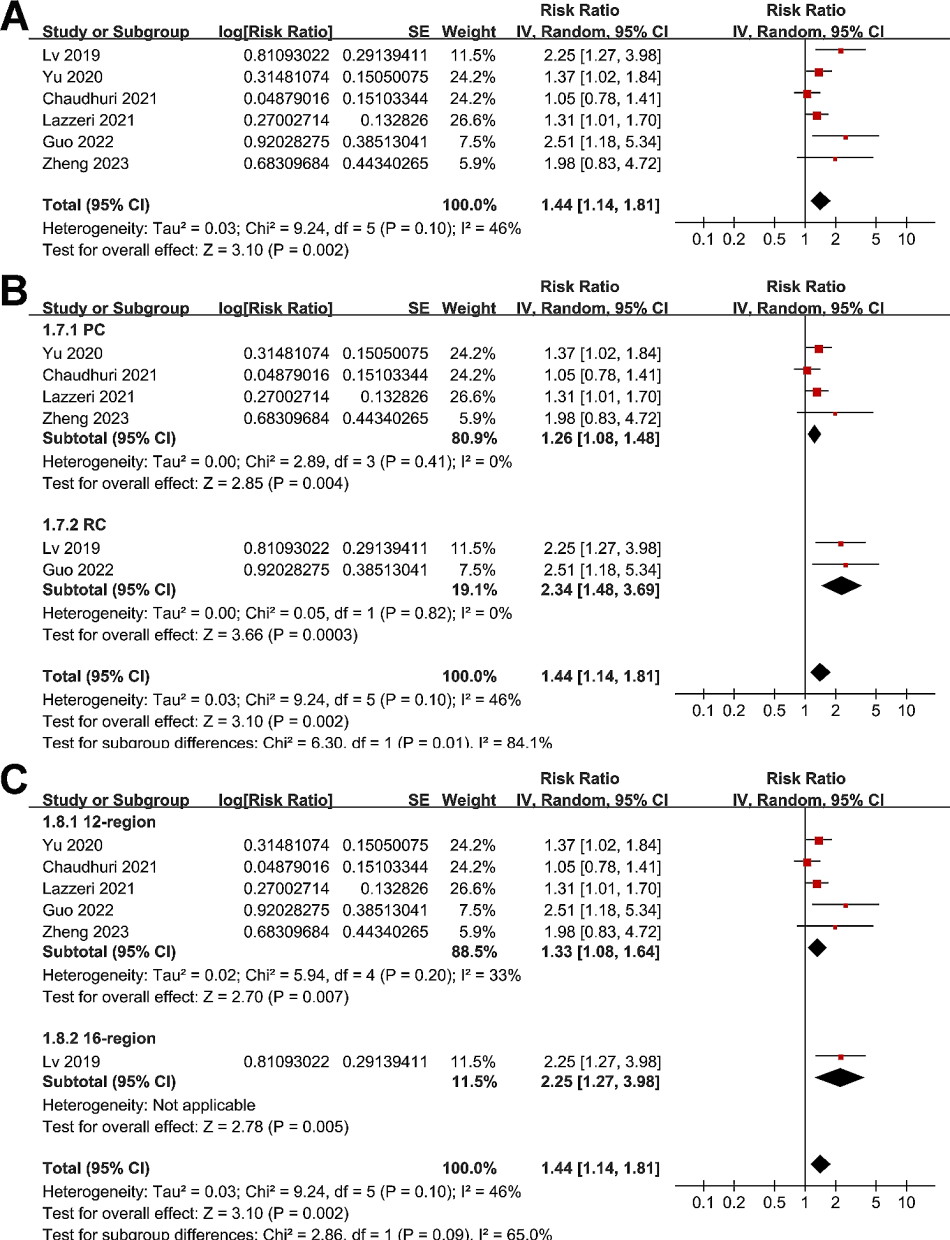
Forest plots for the meta-analysis of the association between LUS score at admission and in-hospital mortality of patients with ARDS; **(A)**, overall meta-analysis; **(B)**, subgroup analysis according to study design; and **(C)**, subgroup analysis according to methods for evaluating LUS score

**Fig. 5:**
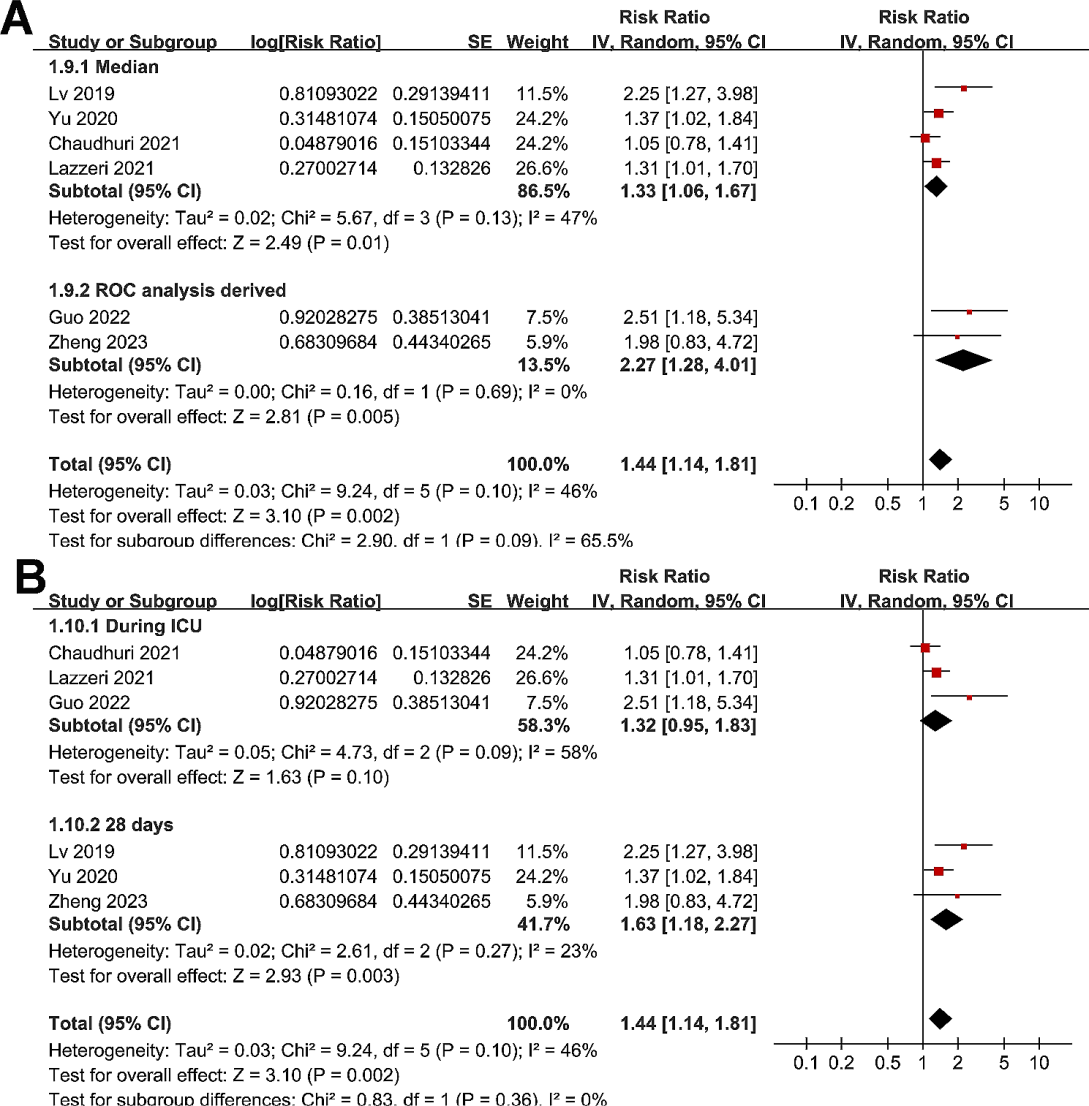
Forest plots for the meta-analysis of the association between LUS score at admission and in-hospital mortality of patients with ARDS; **(A)**, subgroup analysis according to the cutoff of LUS score; and **(B)**, subgroup analysis according to follow-up durations

Meta-analysis of six studies [18, 20, 22, 23, 25, 26] showed that a high LUS score at admission was associated with a higher risk of in-hospital mortality of patients with ARDS (RR: 1.44, 95% CI: 1.14 to 1.81, p = 0.002; Fig. 4A) with moderate heterogeneity (I^2^ = 46%). Sensitivity analysis by excluding one study at a time showed consistent results (RR: 1.33 to 1.55, p all < 0.05). In addition, subgroup analysis showed that the association may be stronger in retrospective studies as compared to prospective studies (RR: 2.34 versus 1.26, p = 0.01; Fig. 4B), which fully explained the source of heterogeneity. Moreover, subgroup analyses showed similar results in studies of LUS score analyzed with 12-region or 16-region method (p for subgroup difference = 0.09, Fig. 4C), in studies using median or ROC derived cutoffs for LUS score (p for subgroup difference = 0.09, Fig. 5A), and in studies reporting mortality during ICU or within 1-month hospitalization (p for subgroup difference = 0.36, Fig. 5B).

### Publication bias

**Fig. 6:**
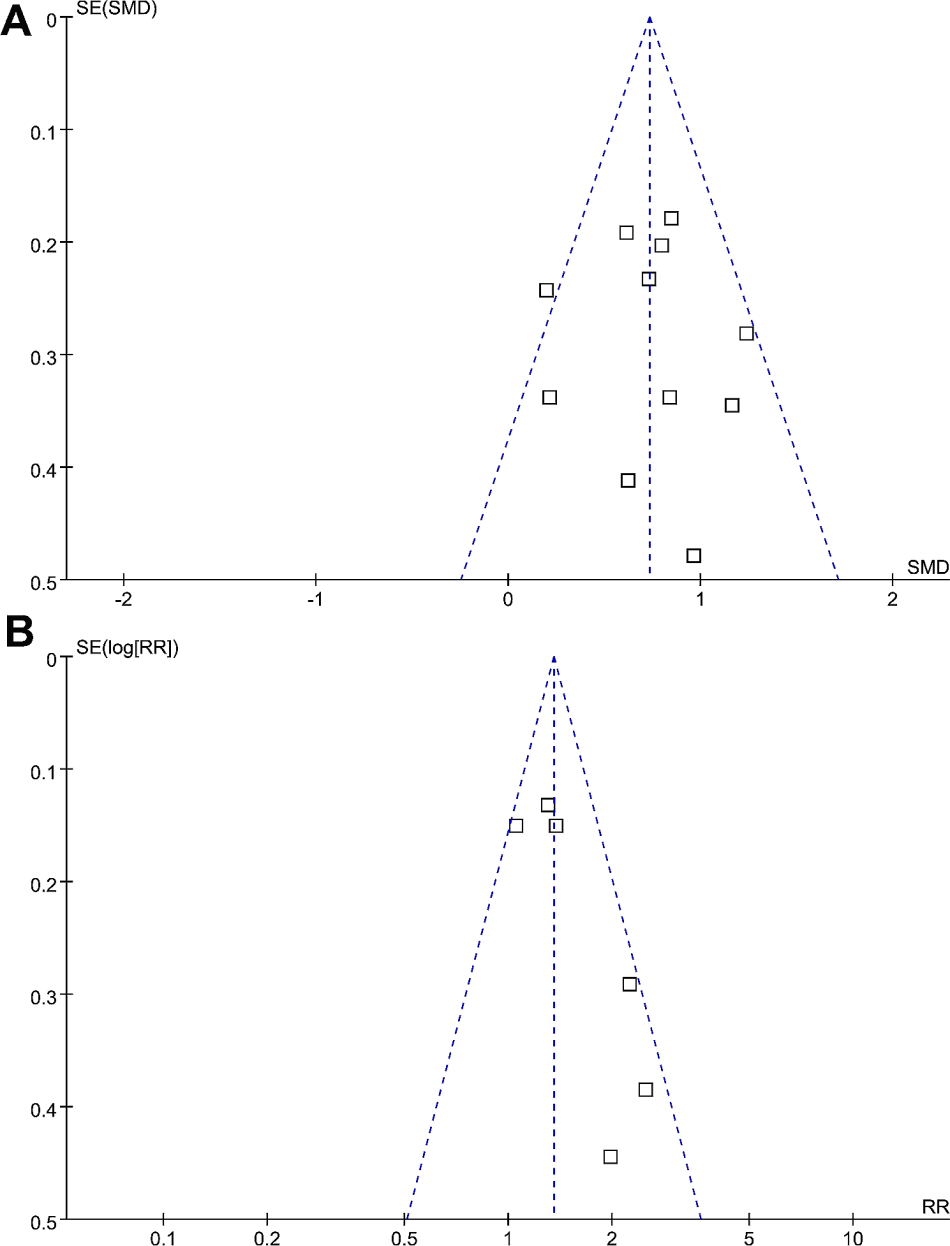
Funnel plots for the publication biases underlying the meta-analyses; **(A)**, funnel plots for the meta-analysis of the difference of LUS score at admission between non-survivors and survivors of ARDS patients; and **(B)**, funnel plots for the meta-analysis of the association between LUS score at admission and in-hospital mortality of patients with ARDS

The funnel plots for the meta-analyses of the difference of LUS scores between non-survivors and survivors and RR for the association between LUS scores and in-hospital mortality of patients with ARDS are shown in Fig. 6A and B. Based on visual examination, the plots are symmetrical, suggesting low publication biases. Additionally, Egger’s regression tests indicated a low likelihood of publication biases (p = 0.81 and 0.29).

## Discussion

This systematic review and meta-analysis integrated the results of 13 cohort studies and showed that a high LUS score at admission may be associated with an increased risk of in-hospital mortality of adult patients with ARDS. Subsequent sensitivity analysis showed consistent results by excluding one study at a time, and in studies of patients with ARDS with the etiology of infection. Moreover, subgroup analysis showed similar results in prospective and retrospective studies, in studies of different methods and cutoff values for evaluating LUS scores, and in studies of different follow-up durations. Taken together, results of the meta-analysis suggested that a high LUS score at admission may be a predictor of in-hospital mortality of adult patients with ARDS.

To the best of knowledge, no meta-analysis has been published regarding the association between LUS score at admission and the in-hospital mortality in patients with ARDS. Ultrasound possesses numerous advantages, including its sterilization convenience, affordability, and lack of radiation [7]. In recent times, LUS has emerged as a precise diagnostic instrument for respiratory ailments [36]. Particularly for patients afflicted with critical conditions like ARDS, LUS can be conducted at the patient’s bedside, thereby mitigating the hazards associated with patient transportation [36]. Furthermore, LUS allows for repetitive examinations, rendering it an exceptional monitoring tool. A sonographer administers the designated LUS protocol and subsequently calculates the LUS score, which is determined by summing the scores assigned to each lung region examined through the measurements of lung aeration loss [37]. Although the protocol of LUS score measurement may be varied, which mainly include the 8-region (0 ~ 24 points), 12-region (0 ~ 36 points), and 16-region (0 ~ 48 points) methods, it has been suggested that the efficacy of these protocols may be similar [38, 39]. Our subgroup results according to the different protocols of LUS score measuring methods also showed consistent results. The mechanisms underlying the association between a high LUS and increased risk of in-hospital mortality of patients with ARDS may be multifactorial. In general, a higher LUS score is indicative of a more severe distribution of extravascular lung water, which indirectly hinders the oxygenation of blood [16]. Furthermore, a higher LUS score in patients diagnosed with ARDS has been found to be correlated with various clinical variables that are associated with a poor prognosis, such as the ratio of arterial oxygen partial pressure to fractional inspired oxygen (PaO_2_/FiO_2_), serum lactate acid levels, and Sequential Organ-Failure Assessment score [18]. Another study conducted on patients with ARDS demonstrated that the LUS score measured at the conclusion of a 60-minute spontaneous breathing trial could serve as a predictor of post-extubation distress, suggesting that a higher LUS score may also be linked to delayed extubation [40]. Furthermore, a recent study has demonstrated a significant correlation between a higher LUS score and patient-reported dyspnea at rest and exertion in individuals who have survived ARDS following their stay in the ICU. These findings imply that, in addition to its ability to predict short-term prognosis, the LUS score may serve as an indicator of the long-term functional status of ARDS patients [41]. From a clinical perspective, results of the meta-analysis support an important role of LUS score for the prognostic evaluation of patients with ARDS. A high LUS score at admission may suggest a worse pulmonary status, which requires timely intensive evaluation with other possible biochemical and imaging examinations, and also an intensive respiratory support. However, further research is required to ascertain the precise role of the LUS score in the management of individuals with ARDS.

The strengths of the meta-analysis include extensive literature search in four databases, performing two independent meta-analyses to confirm the association between LUS score and in-hospital mortality of ARDS, and conducting multiple sensitivity and subgroup analysis to validate the robustness of the findings. However, this study is subject to certain limitations. First, 11 of the included 13 studies were performed in China, and results of the meta-analysis should better be confirmed in large-scale prospective studies from other countries. Second, the optimal protocol and cutoff value for determining the high LUS score at admission in patients with ARDS remain to be determined. In addition, LUS evaluation for ARDS is based on a scoring system according to the regionally presentation of well separated B lines (1 point), coalescent B lines (2 points), and lung consolidation (3 points) [42]. It has to be mentioned that B lines are the product of an artifact of ultrasonic beam reverberation against interfaces of different consistency [43]. Although B lines may be generally caused by extravascular lung water in patients with ARDS, identifying the varying pathophysiological conditions (such as fibrosis) that result in subtle differences in B line appearance is impossible based on LUS alone [43]. Moreover, although multivariate analyses were performed in some of the included studies when the association between LUS and in-hospital mortality was determined, we could not exclude the possibility that there are some other clinical factors that may confound this association. For example, ARDS is a multisystem disease which may involve the dysfunction of other organs besides the lung, and the severity of dysfunctions of these organs could affect the mortality of the patients. Studies are needed in the future to determine if the association between LUS score at admission and the mortality of patients with ARDS is consistent in patients with and without multi-organ dysfunction. Finally, it remains unknown whether incorporating LUS score could improve the predictive efficacy of current prognostic evaluation models for patients with ARDS.

## Conclusions

In conclusion, results of our findings suggest that a high LUS score at admission may be associated with a high risk of in-hospital mortality of patients with ARDS. In view of the multiple advantages of LUS for critically ill patients such as cost-effectiveness, expeditiousness, and bedside accessibility, these findings support the use of LUS score for the severity evaluation and mortality prediction in patients with ARDS.

## Data Availability

The original contributions presented in the study are included in the article, further inquiries can be directed to the corresponding author.
